# Projections of the effects of global warming on the disease burden of ischemic heart disease in the elderly in Tianjin, China

**DOI:** 10.1186/s12889-019-7678-0

**Published:** 2019-11-06

**Authors:** Jing Huang, Qiang Zeng, Xiaochuan Pan, Xinbiao Guo, Guoxing Li

**Affiliations:** 1 Department of Occupational and Environmental Health Sciences, Peking University School of Public Health, Beijing, China; 2 Tianjin Centers for Disease Control and Prevention, 38 Xueyuan Road, Haidian District, Beijing, 100191 China

**Keywords:** Global warming, The elderly, Ischemic heart disease, Years of life lost, Projection

## Abstract

**Background:**

Ischemic heart disease (IHD) is one of the leading causes of deaths worldwide and causes a tremendous disease burden. Temperature is an important environmental determinant among the many risk factors for IHD. However, the emerging temperature-related health risks of IHD in the elderly is limited because of the lack of estimates that integrate global warming and demographic change.

**Methods:**

Data on daily IHD deaths in the elderly aged ≥65 years and meteorological conditions were collected in Tianjin, a megacity of China, from 2006 to 2011. First, the baseline relationship between the temperature and years of life lost (YLL) from IHD was established. Then, future assessments were performed in combination with temperature projections for 19 global-scale climate models (GCMs) under 3 representative concentration pathways (RCPs) for the 2050s and 2070s.

**Results:**

Increased YLL from IHD in the elderly was found to be associated with future ambient temperatures. The annual temperature-related YLL from IHD in the 2050s and 2070s were higher than the baseline. For instance, increases of 4.5, 14.9 and 38.3% were found under the RCP2.6, RCP4.5 and RCP8.5 scenarios, respectively, in the 2070s. The most significant increases occurred in warm season months. The increase in heat-related YLL will not be completely offset, even with the 25% adaptation assumed. When considering demographic change, the temperature-related disease burden of IHD in the elderly will be exacerbated by 158.4 to 196.6% under 3 RCPs in the 2050s and 2070s relative to the baseline.

**Conclusions:**

These findings have significant meaning for environmental and public health policy making and interventions towards the important issue of the health impacts of global warming on the elderly.

## Background

Ischemic heart disease (IHD) is characterized by myocardial ischemia caused by the narrowing of coronary vessels, which supply blood to the heart, and it represents one of the leading causes of deaths worldwide and causes a tremendous disease burden [1]. During the past two decades, the disease burden of IHD has markedly increased in China, with the age-standardized mortality rate increased by 20.6% from 1990 to 2017, and IHD has already become the second leading cause of death in China and was surpassed only by stroke in 2017 [2]. Epidemiological studies have demonstrated that temperature is an important environmental determinant among the many risk factors for IHD, with both extremely cold and heat associated with increased mortality risk from IHD [3, 4].

Global climate change is one of the most serious environmental health problems currently faced by human populations [5]. The Intergovernmental Panel on Climate Change Fifth Assessment Report (IPCC AR5) highlighted that the global mean surface temperature on earth is projected to rise in the future [6]. Many countries worldwide have experienced tremendous burdens of heat-related and cold-related deaths under the current climate patterns [7–9], and global climate change will likely exacerbate such risks [10, 11]. Thus, creating projections of the temperature-related disease burden of IHD for future climate change scenarios is important for the development of environmental and public health policies to mitigate the health effects of extreme temperatures. However, evidence in this field is rare.

Aging has also emerged as a significant social and public health challenge. The elderly population will increase rapidly in the twenty-first century according to the United Nations (UN) projection [12]. Considering the elderly are more vulnerable to temperature-related health risks [13–15], this demographic change may lead to an increased disease burden from IHD in combination with extreme temperature exposure. Nevertheless, our understanding of the emerging temperature-related health risks of IHD in the elderly is limited because of the lack of estimates that integrate global warming and demographic change [16].

In addition, based on the data from the National Bureau of Statistics of China, the population aged ≥65 years old in China has already reached 158 million in 2017, which accounted for 11.4% of the total population. This indicated China has already entered an “aging society” according to the definition by UN, and this trend is still accelerating [17]. Thus, we selected the 65 and older group as the target population to evaluate the effects of global warming on the elderly population whose number will increase rapidly in the next decades.

Previous studies have used mortality as the main health outcome to assess the health impacts of temperature, and the influence of age at death was not taken into account [3, 4, 18]. Years of life lost (YLL), which is an indicator of disease burden, accounts for both premature death and life expectancy at death [19]. However, the availability of studies on the relationships between temperature and YLL from IHD in the elderly is rare, and projections have been insufficient up to now.

The effects of global warming on the disease burden of IHD in the elderly were estimated in a megacity of China in this study. Different climate change scenarios and demographic change in the elderly were considered in the projections. Considering the tremendous disease burden of IHD and its increasing trend in China, our study will provide important information for environmental and public health interventions aimed at reducing the temperature-related disease burden of IHD in an ageing population under global warming scenarios in the future.

## Methods

### Study area

The study area is Tianjin, the third largest municipality in China. It has a population of approximately 12.9 million, with the elderly (age ≥ 65 years) accounting for approximately 8.52% at baseline (Tianjin Statistical Information Site, 2006–2011, http://stats.tj.gov.cn/Category_29/Index.aspx). Tianjin has a typical temperate climate characterized by hot, rainy summers and cold, dry winters.

### Data collection

Daily mortality data on IHD in the elderly (age ≥ 65 years) from Jan 1st 2006 to Dec 31st 2011 in Tianjin were collected from Death Registration and Reporting System of the Chinese Centre for Disease Control and Prevention. The data were include all the residents of Tianjin, and the mortality data were representative of the study area. The causes of IHD were classified and coded according to the International Classification of Disease, 10th version (IHD: I20-I25). Data permission was obtained and the study was approved by the Ethics Committee/Institutional Review Board of Peking University Health Science Center.

Indicator of YLL was used in this study. The method to calculate YLL was used in previous studies as the follow equation [19, 20].

$$ \mathrm{YLL}=\sum Yi\times Li $$

where *Yi* is the death number for a specific age group *i*, and *Li* is the remaining life expectancy for a specific age group *i*.

First, we matched each person’s age at death to the World Health Organization (WHO) standard life table (Additional file 1: Table S1). Then, the daily YLL of the elderly from IHD in our study area was estimated by summing the YLL of all individuals who aged ≥65 years and died from IHD on the same day.

Daily meteorological data, including the daily relative humidity and maximum temperature, were obtained from the Tianjin Meteorological Bureau, which were match with the daily mortality data of IHD during the study period from 2006 to 2011. The average daily concentrations of particulate matter with aerodynamic diameter ≤ 10 μm (PM_10_) were also collected from the Tianjin Environmental Monitoring Centre to allow for the evaluation of the confounding effects of air pollutants.

### Statistical analyses

First, the baseline temperature-YLL relationships from 2006 to 2011 were established, then future assessments were made in combination with future temperature projections.

Distributed lag non-linear models (DLNMs) were used to measure the non-linear and delayed effects of temperature on YLL from IHD during the baseline period [21]. Seasonal and long-term trend were adjusted using a natural cubic spline function with 7 degrees per year, and day of the week as a categorical variable. The daily PM_10_ and relative humidity were adjusted using a natural cubic spline with 3 degrees of freedom. A natural cubic B-spline basis with 5 degrees of freedom for temperature and a maximum lag of 15 days between temperature and YLL with 5 degrees of freedom were chosen in the baseline analysis. Optimal temperature (OT) was used as a reference to calculate the temperature-related YLL related to high or low temperatures. The OT was determined according to the exposure-response curve for temperature in YLL for IHD during the baseline period.

Temperature projections were made according to Representative Concentration Pathways (RCPs) reported in the IPCC AR5. Three RCPs scenarios were selected in our analysis, including RCP2.6, RCP4.5 and RCP8.5, which represent the mild, the medium and the high emission scenarios. The future daily temperatures for periods of 2046 to 2065 centred on 2050s, and 2061 to 2080 centred on 2070s, were developed from 19 global-scale climate models (GCMs) from the World Climate Research Programme (WCRP) Coupled Model Intercomparison Project Phase 5 (CMIP 5) multi-model dataset (Additional file 1: Table S2).

As for the temperature calibration, we used method as follows: it started with the projected change in a weather variable (i.e. maximum temperature in June). This is computed as the (absolute or relative) difference between the output of the GCM run for the baseline years and for the target years (e.g. 2050s–2070s). These changes are then added to the observed baseline to create the projected temperature in a given year for a specific emission scenario (in this case the WorldClim Database, http://www.worldclim.org/downscaling). Through this method, we could capture the trend of temperature change in future projection. And this approach was also used in previous study [22].

The projection of temperature-related YLL from IHD in the elderly was calculated by integrating the temperature projections under different RCPs with baseline exposure-response relationships. The projections and changes of annual heat-related, cold-related and total temperature-related YLL were calculated.

Furthermore, adaptation of the population was taken into consideration because people could adapt to warmer climatic conditions through a number of measures [23]. A 25% acclimatization factor was assumed according to the previous study of a U.S. city, which reported that excess mortality related with heat reduced by approximately 25%, indicating population adaptation to heat in recent decades through increased using of air conditioning, greater awareness of the risks by high temperature, and introduction of heat-warning systems, etc. [24].

Considering the temperature-YLL relationship may not remain stable over time due to population adaptation, we modelled the adaptation by shifting the OT and shape of temperature-YLL curves [25]. The model combining the absolute threshold shift of OT with the reduction in the slope of the heat exposure-response function was used in this study because it may balance the uncertainty among adaptation models, climate models and emissions [26]. Previous studies have suggested that OT could continue to rise with increasing temperature due to adaptation [26, 27]. Absolute threshold shift of OT is a popular method for modeling adaptation [26], and applying a shift in absolute threshold of OT between 1.0 °C and 4.0 °C in future is recommended because this is broadly within the range of shifts in threshold temperature observed in previous epidemiological studies [27–29]. Thus, we conservatively estimated that the OT will increase by 1.0 °C in 2050s, and by 1.2 °C in 2070s relative to the baseline [30].

Demographic change was also taken into account. Based on the low, medium and high variant scenarios of population growth among the population aged 65 years and above in China employed by the UN, this population size will be 3.1 and 3.2 times greater than the baseline population in 2010 by the 2050s and 2070s, respectively [12].

Sensitivity analyses were performed to test whether the results were robust to changes in the parameters in the model, including using 4 degrees of freedom of relative humidity, and a natural cubic B-spline with 5 degrees of freedom for temperature and a maximum lag of 15 days between temperature and YLL with 6 degrees of freedom.

R software was used to perform all analyses (version 3.3.3, http://www.R-project.org/).

## Results

### Descriptive statistics of deaths, YLL and temperature data

A total of 68,485 IHD deaths were identified during the period from 2006 to 2011 in Tianjin, and the corresponding YLL were 585,717 years. The mean daily death counts for IHD was 31.3, and the corresponding mean daily YLL was 267.3 years over the study period. The mean daily maximum temperature was 18.0 °C, with a range from − 9.2 °C to 38.8 °C. The average daily maximum temperatures under the RCP2.6, RCP4.5 and RCP8.5 were projected to increase by 1.4 °C, 1.7 °C and 2.4 °C in the 2050s, respectively, and by 1.4 °C, 2.3 °C and 3.8 °C in the 2070s, respectively, compared with the baseline period (Additional file 1: Table S3).

### Baseline associations between temperature and YLL from IHD

**Fig. 1:**
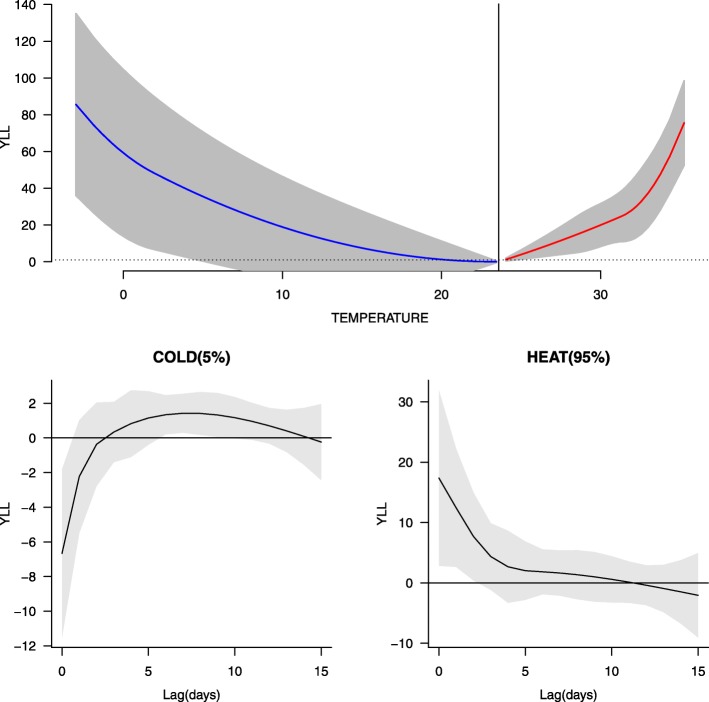
Effects of temperature on years of life lost from ischemic heart disease in the elderly in Tianjin, China, from 2006 to 2011. The upper panel shows the exposure-response curve between the daily maximum temperature and years of life lost. The bottom panel shows the delayed effects of temperature on years of life lost by lag at cold and heat temperature. The solid line and the grey area show the mean and 95% confidence interval, respectively. -0.3 °C and 33.0 °C are the 5th and 95th percentiles of the daily maximum temperature used to represent cold and heat temperatures, respectively

The exposure-response curve between the daily maximum temperature and YLL from IHD in the elderly is shown in the upper panel of Fig. 1. The curve was approximately U-shaped and presented an OT of 23.6 °C. The results of sensitivity analyses indicated that the OT was stable to the specification of the parameters in the model (Additional file 1: Figure S1). Temperatures higher or lower than this temperature resulted in increased YLL from IHD. Low temperature was considered lower than the OT, and high temperature was defined as temperature higher than the OT. In general, cold effects were associated with longer-lasting risks than heat effects. The peak of the cold effects occurred 7 days after exposure and declined gradually over the following 5 days, whereas the greatest heat effects appeared on the 1st day of exposure and decreased rapidly the following day. To avoid an overestimation of cold and heat effects, the cumulative effects of lag0-lag12 and lag0-lag2 for cold temperatures and heat temperatures were selected in the main analysis, respectively.

The baseline annual heat-related, cold-related and total temperature-related YLL from IHD in the elderly was 3200 years, 5508 years and 8708 years, respectively.

### Projected temperature-related YLL from IHD

The projected attributable proportions of YLL from IHD in the elderly caused by nonoptimal temperatures under the 3 RCPs are presented in Additional file 1: Figure S2. In the baseline period, heat-related and cold-related YLL accounted for 3.3 and 5.6% of the total IHD YLL, respectively. Under the global warming scenarios, heat-related YLL showed an increasing trend, while the cold-related YLL showed a decreasing trend. For example, under RCP8.5, the proportion of heat effects increased to 9.0%, while the proportion of cold effects was reduced to 3.4%.

The total annual temperature-related YLL from IHD in the 2050s and 2070s were higher than the baseline values and increased by 6.3, 7.6 and 15.4% in the 2050s, respectively, and by 4.5, 14.9 and 38.3% in the 2070s, respectively, under the 3 RCPs (Table 1). Heat-related YLL from IHD will increase by 46.9, 55.1, and 87.3% in the 2050s, respectively, and by 43.0, 85.3 and 174.2% in the 2070s. The changes of annual heat-related, cold-related and total annual temperature-related YLL in the 2050s and 2070s for each GCM under the 3 RCPs are presented in Additional file 1: Table S4 and Table S5.

**Table 1:** Averaged projections of annual heat-related, cold-related and total temperature-related years of life lost from ischemic heart disease and percentage changes in the 2050s, 2070s for 19 global-scale climate change models under 3 representative concentration pathways compared with baseline 2006–2011, Tianjin, China

Periods	Projection and Change (%)	RCP2.6			RCP4.5			RCP8.5		
		Heat^a^	Cold^b^	Total^c^	Heat^a^	Cold^b^	Total^c^	Heat^a^	Cold^b^	Total^c^
2050s	Projection	4702	4556	9258	4964	4410	9374	5995	4058	10,053
	Change (%)	46.9%	−17.3%	6.3%	55.1%	−20.0%	7.6%	87.3%	−26.3%	15.4%
2070s	Projection	4567	4526	9102	5931	4072	10,003	8773	3271	12,044
	Change (%)	43.0%	−17.8%	4.5%	85.3%	−26.1%	14.9%	174.2%	−40.6%	38.3%

^a, b, c^Percentage change relative to annual baseline heat-related and cold-related, and total temperature-related years of life lost from ischemic heart disease is 3200 years, 5508 years, and 8708 years, respectively. The unit of years of life lost projection is year

**Fig. 2:**
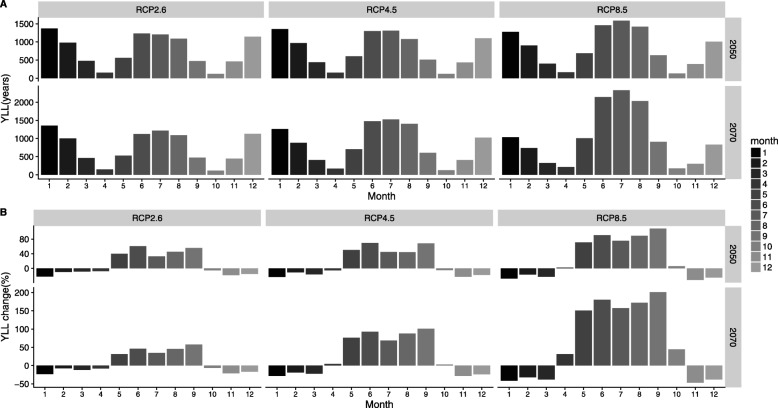
**a** Average projected absolute monthly temperature-related years of life lost from ischemic heart disease in the elderly in the 2050s and 2070s for 19 global-scale climate models under 3 representative concentration pathways (RCPs). **b** Average projected percent change (%) of monthly temperature-related years of life lost from ischemic heart disease in the 2050s and 2070s for 19 global-scale climate models under 3 representative concentration pathways (RCPs), compared with the baseline period from 2006 to 2011

Projections of the monthly levels of temperature-related YLL from IHD in the elderly are presented in Fig. 2a. In general, among the 12 months, a large percentage increase occurred in the warm season months (from May to September), while a relatively small reduction appeared in the cool season months (from November to March). Regarding the high emission scenario of RCP8.5, compared with the baseline period from 2006 to 2011, the most significant increases appeared in September, with increment percentages of 108.7 and 201.3% in the 2050s and 2070s, respectively. The largest reduction occurred in November, with reduction percentages of 31.8 and 47.1% in the 2050s and 2070s, respectively, under RCP8.5, as shown in Fig. 2b.

### Scenarios of adaptation to future global warming

**Fig. 3:**
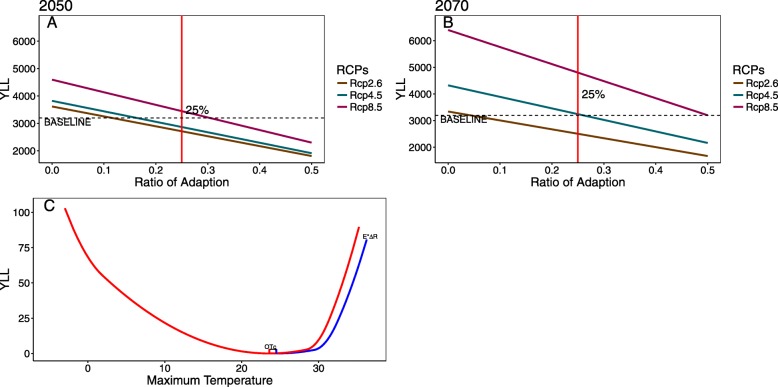
**a** Projections of years of life lost from ischaemic heart disease in the elderly against future heat temperature adaptations under the 3 representative concentration pathways (RCPs) in the 2050s. **b** Projections of years of life lost from ischaemic heart disease in the elderly against future heat temperature adaptations under the 3 representative concentration pathways (RCPs) in the 2070s. In (**a**) and (**b**), the percent adaptation necessary to offset the projected heat-related years of life lost is defined where the slopes cross the horizontal dotted line. The vertical solid red line represents the 25% adaptation assumed in the study. **c** Projections of years of life lost from ischaemic heart disease in the elderly according to scenarios of adaptations to heat temperatures in Tianjin, China. The blue curve indicates the percent reductions in the effects of heat temperatures due to future adaptation by E*R in the temperature space (0 < R ≤ 0.5), where E represents the effects of heat temperature; R is the ratio of adaptation; and OTc represents the optimal temperature increase. OT is assumed to increase by 1.0 °C and 1.2 °C in the 2050s and 2070s relative to the baseline, respectively

Scenarios of adaptation to future global warming were accounted for in this study. Heat-related effects will be reduced when considering adaptation. The projections of YLL from IHD in the elderly against adaptations to future global warming under the 3 RCPs in the 2050s and 2070s are shown in Fig. 3. The percent adaptation necessary to offset the projected heat-related YLL was 12, 17 and 31% in the 2050s for RCP2.6, RCP4.5 and RCP8.5, respectively, whereas these values were 5, 26 and 50% in the 2070s, respectively. Considering the 25% adaptation assumed in our study, the increment in heat-related YLL will not be completely offset under RCP 8.5 in the 2050s or in RCP4.5 or RCP8.5 in the 2070s.

### Projections considering population growth in the elderly

When taking population growth in the elderly aged 65 and above into account, the projected temperature-related YLL from IHD will be further amplified. The projected heat-related, cold-related and total temperature-related YLL when considering both population growth in the elderly and adaptation were shown in Table 2. Under the UN population growth scenario, the total temperature-related YLL from IHD increased rapidly in the 2050s and 2070s compared with that at baseline, with percentage increments of 158.8, 159.0 and 167.2% observed in the 2050s, respectively, and 158.4, 168.8 and 196.6% observed in the 2070s, respectively, under the 3 RCPs, even taking into consideration the 25% adaptation used in this study.

**Table 2:** Projections of heat-related, cold-related and total temperature-related years of life lost from ischemic heart disease when considering both population growth in the elderly and adaptation

Periods	RCP2.6			RCP4.5			RCP8.5		
	Heat (%)^a^	Cold (%)^b^	Total (%)^c^	Heat (%)^a^	Cold (%)^b^	Total (%)^c^	Heat (%)^a^	Cold (%)^b^	Total (%)^c^
2050s	8409(162.8%)	14,124(156.4%)	22,532(158.8%)	8888(177.8%)	13,670(148.2%)	22,558(159.0%)	10,685(233.9%)	12,580(128.4%)	23,265(167.2%)
2070s	8018(150.6%)	14,483(162.9%)	22,501(158.4%)	10,375(224.2%)	13,029(136.5%)	23,404(168.8%)	15,362(380.1%)	10,469 (90.1%)	25,831(196.6%)

^a, b, c^Percentage change relative to annual baseline heat-related and cold-related, and total temperature-related years of life lost from ischemic heart disease is 3200 years, 5508 years, and 8708 years, respectively

## Discussion

This study estimated future temperature-related YLL from IHD in the elderly in Tianjin, China, in the 2050s and 2070s. To the best of our knowledge, this is the first study that has estimated the effects of global warming on the disease burden of IHD in the elderly using the 19GCMs as well as the RCP scenarios and considering ageing population growth over the twenty-first century.

We found substantial increases in heat-related IHD YLL but relatively slight decreases in cold-related IHD YLL under future global warming scenarios. The increments in heat-related IHD YLL will not be offset by the reduction in cold-related IHD YLL. The finding indicated an increasing disease burden from IHD in the elderly caused by rising temperatures in the future. And the results were consistent with previous studies which also found the increase in the heat-related deaths is unlikely to be offset by the decrease in cold-related deaths [5, 31].

Exposure to heat may induce profound physiological changes, including increased blood viscosity and cardiac output, which can lead to dehydration, hypotension, increased surface blood circulation and even endothelial cell damage [32]. These changes can cause haemoconcentration and overload the function of the heart.

A study conducted in Beijing, China pointed out that the heat-related cardiovascular deaths in the whole population are projected to increase by 135% under RCP8.5 in the 2080s compared with the baseline [33]. While in our study, more than 150% increase was found under RCP8.5 in the 2070s compared with the baseline in the elderly, which indicated more pronounced effect was observed in the elderly.

Considering that aging reduces the ability to thermoregulate, disrupts homoeostasis and the elderly were more likely to living alone, the elderly were more susceptible to the adverse effects of extreme temperatures [13]. Thus more attention should be paid to the temperature-related IHD disease burden in the elderly under global warming scenarios.

Monthly analyses showed that large percent increases occurred mainly in the warm season months from May to September. Considering global climate change is likely to produce more frequent, more intense and longer-lasting heatwaves, the elderly will experience additional stress in the warmer months. These findings will provide important information for environmental and public health resource distribution for IHD according to the monthly change patterns in future.

Because people may adapt to warming climates by increasing their use of air conditioning, improving building design and city planning, implementing early warning systems and changing behaviours [31], human adaptation to a warming climate was also considered in this study. However, the increment in heat-related YLL will not completely offset under the medium emission scenario in the 2070s or the high emission scenario in the 2050s and 2070s, even with an assumed 25% adaptation.

Demographic change is another important factor that should be considered when exploring the health impacts of climate change. Nevertheless, most previous studies have focused only on climate change and ignored possible demographic change [18, 34]. According to UN projections, the world population is ageing rapidly [12]. Therefore, if population growth in the elderly is not properly considered, the temperature-related health burden will be greatly underestimated. Compared with the baseline, the total temperature-related YLL in the elderly will increase rapidly in the 2050s and 2070s by 158.4 to 196.6% in Tianjin, China.

There are several strengths of this study. First, to our knowledge, it is the first time to explore the temperature-related disease burden of IHD in the elderly under future climate change scenarios. These data will provide scientific evidence for policy making on the important issue of global warming on the elderly. Second, monthly analyses were conducted to explore changes in temperature-related YLL from IHD by different months, which can be crucial for resources allocation for IHD according to different monthly patterns. Third, the disease burden was estimated using the indicator of YLL, which is an informative measurement for quantifying premature mortality [35]. In addition, projections were made that accounted for both the adaptation of the population to the warming climate and demographic change in the elderly.

This study also had certain limitations. First, the data in our study were limited to one megacity with typical temperate climate, thus generalizing the results to other geographic areas should be performed with caution. Second, temperature data from the fixed sites rather than the individual data were used, but measurement errors may bias the results towards the null hypothesis [36]. Third, climate change is likely to produce more frequent and more intense heatwaves. Because additional effects of heat waves may be observed [37], our results may have underestimated the future IHD YLL in the elderly due to climate change.

## Conclusions

Our study projected the effects of global warming on the disease burden of IHD in the elderly using the indicator of YLL in Tianjin, a megacity of China. The annual temperature-related YLL from IHD in the 2050s and 2070s were increased compared with the baseline in 2006–2011, and monthly analyses showed that large increases occurred mainly in the warm season months. When considering demographic change, the temperature-related disease burden of IHD in the elderly will be exacerbated by 158.4 to 196.6% under 3 RCPs in the 2050s and 2070s relative to baseline, even with 25% adaptation taken into consideration. These findings indicated that more environmental and public health policy making and interventions measures should be undertaken to alleviate the impacts of global warming on the disease burden of IHD in the elderly.

## Supplementary information

**Additional file 1: Table S1.** World Health Organization (WHO) standard life table for years of life lost. **Table S2.** Global-scale Climate Models (GCMs) used in this study. **Table S3.** Daily death counts and years of life lost from ischemic heart disease, baseline environmental conditions from 2006 to 2011 in Tianjin, China, and projected temperatures under 3 representative concentration pathways in the 2050s and 2070s. **Table S4.** The changes of annual heat-related, cold-related, and net total temperature-related years of life lost from ischemic heart disease for each global-scale climate model under 3 representative concentration pathways (RCPs) in 2050s. **Table S5.** The changes of annual heat-related, cold-related, and net total temperature-related years of life lost from ischemic heart disease for each global-scale climate model under 3 representative concentration pathways (RCPs) in 2070s. **Figure S1.** Sensitivity analyses of exposure-response curve between the daily maximum temperature and years of life lost from ischemic heart disease in the elderly in Tianjin, China, from 2006 to 2011. a. Using 4 degrees of freedom of relative humidity in the model (the optimal temperature is 23.7 °C) b. A natural cubic B-spline basis with 5 degrees of freedom for temperature and a maximum lag of 15 days between temperature and YLL with 6 degrees of freedom in the model (the optimal temperature is 23.4 °C). **Figure S2.** Projected attributable proportions of heat-related, cold-related and total temperature-related years of life lost from ischaemic heart disease in the elderly for 19 global-scale climate models under 3 representative concentration pathways (RCPs).

## Data Availability

Data are available for consultation upon request to the corresponding author.

## Abbreviations

CMIP5: Coupled Model Intercomparison Project Phase 5
DLNMs: Distributed lag non-linear models
GCMs: Global-scale climate models
IHD: Ischemic heart disease
IPCC AR5: the Intergovernmental Panel on Climate Change Fifth Assessment Report
OT: Optimal temperature
PM_10_: Particulate matter with aerodynamic diameter ≤ 10 μm
RCPs: Representative concentration pathways
UN: United Nations
WCRP: the World Climate Research Programme
WHO: World Health Organization
YLL: Years of life lost
