# Supplementary material for: Projections of the effects of global warming on the disease burden of ischemic heart disease in the elderly in Tianjin, China
Source: BMC Public Health. 2019 Nov 6;19:1465. doi: 10.1186/s12889-019-7678-0 (PMC6836533; doi:10.1186/s12889-019-7678-0)
Supplement: Supplementary file 1 — Additional file 1: Table S1. World Health Organization (WHO) standard life table for years of life lost. Table S2. Global-scale Climate Models (GCMs) used in this study. Table S3. Daily death counts and years of life lost from ischemic heart disease, baseline environmental conditions from 2006 to 2011 in Tianjin, China, and projected temperatures under 3 representative concentration pathways in the 2050s and 2070s. Table S4. The changes of annual heat-related, cold-related, and net total temperature-related years of life lost from ischemic heart disease for each global-scale climate model under 3 representative concentration pathways (RCPs) in 2050s. Table S5. The changes of annual heat-related, cold-related, and net total temperature-related years of life lost from ischemic heart disease for each global-scale climate model under 3 representative concentration pathways (RCPs) in 2070s. Figure S1. Sensitivity analyses of exposure-response curve between the daily maximum temperature and years of life lost from ischemic heart disease in the elderly in Tianjin, China, from 2006 to 2011. a. Using 4 degrees of freedom of relative humidity in the model (the optimal temperature is 23.7 °C) b. A natural cubic B-spline basis with 5 degrees of freedom for temperature and a maximum lag of 15 days between temperature and YLL with 6 degrees of freedom in the model (the optimal temperature is 23.4 °C). Figure S2. Projected attributable proportions of heat-related, cold-related and total temperature-related years of life lost from ischaemic heart disease in the elderly for 19 global-scale climate models under 3 representative concentration pathways (RCPs). [file 12889_2019_7678_MOESM1_ESM.docx]

**Additional files**

**Projections of the effects of global warming on the disease burden of ischemic heart disease in the elderly in a megacity, China**

**Table S1.** World Health Organization (WHO) standard life table for years of life lost.

**Table S2.** Global-scale Climate Models (GCMs) used in this study.

**Table S3**. Daily death counts and years of life lost from ischemic heart disease, baseline environmental conditions from 2006-2011 in Tianjin, China, and projected temperatures under 3 representative concentration pathways in the 2050s and 2070s.

**Table S4.** The changes of annual heat-related, cold-related, and net total temperature-related years of life lost from ischemic heart disease for each global-scale climate model under 3 representative concentration pathways (RCPs) in 2050s.

**Table S5.** The changes of annual heat-related, cold-related, and net total temperature-related years of life lost from ischemic heart disease for each global-scale climate model under 3 representative concentration pathways (RCPs) in 2070s.

**Figure S1.** Sensitivity analyses of exposure-response curve between the daily maximum temperature and years of life lost from ischemic heart disease in the elderly in Tianjin, China, from 2006-2011.

1. Using 4 degrees of freedom of relative humidity in the model (the optimal temperature is 23.7 °C)
2. A natural cubic B-spline basis with 5 degrees of freedom for temperature and a maximum lag of 15 days between temperature and YLL with 6 degrees of freedom in the model (the optimal temperature is 23.4 °C)

**Figure S2.** Projected attributable proportions of heat-related, cold-related and total temperature-related years of life lost from ischaemic heart disease in the elderly for 19 global-scale climate models under 3 representative concentration pathways (RCPs).

**Table S1.** World Health Organization (WHO) standard life table for years of life lost.

| Age | SEYLL* | Age | SEYLL | Age | SEYLL |
| --- | --- | --- | --- | --- | --- |
| 0 | 91.94 | 35 | 57.15 | 70 | 23.15 |
| 1 | 91 | 36 | 56.16 | 71 | 22.23 |
| 2 | 90.01 | 37 | 55.17 | 72 | 21.31 |
| 3 | 89.01 | 38 | 54.18 | 73 | 20.4 |
| 4 | 88.02 | 39 | 53.19 | 74 | 19.51 |
| 5 | 87.02 | 40 | 52.2 | 75 | 18.62 |
| 6 | 86.02 | 41 | 51.21 | 76 | 17.75 |
| 7 | 85.02 | 42 | 50.22 | 77 | 16.89 |
| 8 | 84.02 | 43 | 49.24 | 78 | 16.05 |
| 9 | 83.03 | 44 | 48.25 | 79 | 15.22 |
| 10 | 82.03 | 45 | 47.27 | 80 | 14.41 |
| 11 | 81.03 | 46 | 46.28 | 81 | 13.63 |
| 12 | 80.03 | 47 | 45.3 | 82 | 12.86 |
| 13 | 79.03 | 48 | 44.32 | 83 | 12.11 |
| 14 | 78.04 | 49 | 43.34 | 84 | 11.39 |
| 15 | 77.04 | 50 | 42.36 | 85 | 10.7 |
| 16 | 76.04 | 51 | 41.38 | 86 | 10.03 |
| 17 | 75.04 | 52 | 40.41 | 87 | 9.38 |
| 18 | 74.05 | 53 | 39.43 | 88 | 8.76 |
| 19 | 73.05 | 54 | 38.46 | 89 | 8.16 |
| 20 | 72.06 | 55 | 37.49 | 90 | 7.6 |
| 21 | 71.06 | 56 | 36.52 | 91 | 7.06 |
| 22 | 70.07 | 57 | 35.55 | 92 | 6.55 |
| 23 | 69.07 | 58 | 34.58 | 93 | 6.07 |
| 24 | 68.08 | 59 | 33.62 | 94 | 5.6 |
| 25 | 67.08 | 60 | 32.65 | 95 | 5.13 |
| 26 | 66.09 | 61 | 31.69 | 96 | 4.65 |
| 27 | 65.09 | 62 | 30.73 | 97 | 4.18 |
| 28 | 64.1 | 63 | 29.77 | 98 | 3.7 |
| 29 | 63.11 | 64 | 28.82 | 99 | 3.24 |
| 30 | 62.11 | 65 | 27.86 | 100 | 2.79 |
| 31 | 61.12 | 66 | 26.91 | 101 | 2.36 |
| 32 | 60.13 | 67 | 25.96 | 102 | 1.94 |
| 33 | 59.13 | 68 | 25.02 | 103 | 1.59 |
| 34 | 58.14 | 69 | 24.08 | 104 | 1.28 |
|  |  |  |  | 105 | 1.02 |

*SEYLL: standard expected years of life lost. (from Global Health Estimates Technical Paper WHO/HIS/HSI/ GHE/ 2013.4)

The study population is the elderly age ≥65 years, thus age≥65 years in the life table was used.

**Table S2.** Global-scale Climate Models (GCMs) used in this study.

| **No** | **Climate Model Acronym** | **code** | **Institute** |
| --- | --- | --- | --- |
| 1 | ACCESS1-0 | AC | Commonwealth Scientific and Industrial Research Organization and Bureau of Meteorology, Australia |
| 2 | BCC-CSM1-1 | BC | Beijing Climate Center, China Meteorological Administration |
| 3 | CCSM4 | CC | National Center for Atmospheric Research |
| 4 | CESM1-CAM5-1-FV2 | CE | Community Earth System Model Contributors |
| 5 | CNRM-CM5 | CN | Centre National de Recherches Météorologiques/ Centre Européen de Recherche et Formation Avancée en Calcul Scientifique |
| 6 | GFDL-CM3 | GF | NOAA Geophysical Fluid Dynamics Laboratory |
| 7 | GFDL-ESM2G | GD | NOAA Geophysical Fluid Dynamics Laboratory |
| 8 | GISS-E2-R | GS | NASA Goddard Institute for Space Studies |
| 9 | HadGEM2-AO | HD | Met Office Hadley Centre (additional HadGEM2­ ES realizations contributed by Instituto Nacional de Pesquisas Espaciais) |
| 10 | HadGEM2-CC | HG | Met Office Hadley Centre (additional HadGEM3­ ES realizations contributed by Instituto Nacional de Pesquisas Espaciais) |
| 11 | HadGEM2-ES | HE | Met Office Hadley Centre (additional HadGEM4­ ES realizations contributed by Instituto Nacional de Pesquisas Espaciais) |
| 12 | INMCM4 | IN | Institute for Numerical Mathematics |
| 13 | IPSL-CM5A-LR | IP | Institute for Numerical Mathematics |
| 14 | MIROC-ESM-CHEM (#) | MI | Japan Agency for Marine-Earth Science and Technology, Atmosphere and Ocean Research Institute  (The University of Tokyo), and National Institute for Environmental Studies |
| 15 | MIROC-ESM (#) | MR | Japan Agency for Marine-Earth Science and Technology, Atmosphere and Ocean Research Institute  (The University of Tokyo), and National Institute for Environmental Studies |
| 16 | MIROC5 (#) | MC | Atmosphere and Ocean Research Institute (The University of Tokyo), National Institute for Environmental Studies,  and Japan Agency for Marine-Earth Science and Technology |
| 17 | MPI-ESM-LR | MP | Max-Planck-Institut für Meteorologie (Max Planck Institute for Meteorology) |
| 18 | MRI-CGCM3 | MG | Meteorological Research Institute |
| 19 | NorESM1-M | NO | Norwegian Climate Centre |

**Table S3**. Daily death counts and years of life lost from ischemic heart disease, baseline environmental conditions from 2006-2011 in Tianjin, China, and projected temperatures under 3 representative concentration pathways in the 2050s and 2070s.

| **Variables** | | **Mean±SD** | **Min** | **25th** | **50th** | **75th** | **Max** |
| --- | --- | --- | --- | --- | --- | --- | --- |
| **Ischemic heart disease** | |  |  |  |  |  |  |
| Death counts | | 31.3±12.9 | 2.0 | 21.0 | 31.0 | 40.0 | 91.0 |
| Years of life lost(years) | | 267.3±110.0 | 16.9 | 181.9 | 268.2 | 342.2 | 726.6 |
| **Environmental conditions** | |  |  |  |  |  |  |
| Daily maximum temperature (℃) | | 18.0±11.3 | -9.2 | 7.5 | 19.9 | 28.4 | 38.8 |
| Daily Relative humidity (%) | | 58.4±18.4 | 15.0 | 44.0 | 60.0 | 73.0 | 95.5 |
| PM_10_ | | 98.5±56.7 | 10.0 | 60.0 | 86.0 | 122.0 | 503.0 |
| **Projections of Tmax** (℃) | |  |  |  |  |  |  |
| 2050s | RCP2.6 | 19.4±11.3 | -5.7 | 8.7 | 21.2 | 30.0 | 39.2 |
|  | RCP4.5 | 19.7±11.2 | -5.6 | 8.9 | 21.6 | 30.1 | 39.3 |
|  | RCP8.5 | 20.4±11.3 | -5.0 | 9.6 | 22.2 | 30.8 | 40.0 |
| 2070s | RCP2.6 | 19.4±11.2 | -5.6 | 8.8 | 21.2 | 29.9 | 39.1 |
|  | RCP4.5 | 20.3±11.2 | -4.9 | 9.6 | 22.1 | 30.7 | 39.8 |
|  | RCP8.5 | 21.8±11.1 | -3.1 | 11.3 | 23.5 | 32.3 | 41.4 |

**Table S4.** The changes of annual heat-related, cold-related, and net total temperature-related years of life lost from ischemic heart disease for each global-scale climate model under 3 representative concentration pathways (RCPs) in 2050s.

| **GCMs** | **RCP2.6** | | | **RCP4.5** | | | **RCP8.5** | | |
| --- | --- | --- | --- | --- | --- | --- | --- | --- | --- |
|  | **Heat^a^** | **Cold^b^** | **Total^c^** | **Heat^a^** | **Cold^b^** | **Total^c^** | **Heat^a^** | **Cold^b^** | **Total^c^** |
| AC | NA | NA | NA | 2194.6 | -1276.2 | 918.3 | 3987.9 | -1478.4 | 2509.5 |
| BC | 1966.6 | -716.9 | 1249.7 | 1817.5 | -1019.8 | 797.7 | 4028.4 | -1267.3 | 2761.1 |
| CC | 932.9 | -823.4 | 109.6 | 1079.2 | -930.6 | 148.6 | 2228.8 | -1259.4 | 969.4 |
| CE | NA | NA | NA | 6922.9 | -1390.8 | 5532.1 | NA | NA | NA |
| CN | 719.3 | -611.6 | 107.7 | 710.7 | -783.4 | -72.7 | 1595.4 | -1064.7 | 530.7 |
| GD | 384.2 | 285.5 | 669.7 | 449.4 | -353.5 | 95.9 | NA | NA | NA |
| GF | 2328.2 | -1587 | 741.2 | 3025.3 | -1509.1 | 1516.2 | 4456.7 | -1958.5 | 2498.2 |
| GS | 563.7 | -836.8 | -273.1 | 964.4 | -1136 | -171.6 | 1057.7 | -1273 | -215.3 |
| HD | 1613.2 | -1060.7 | 552.5 | 914.2 | -1174.2 | -260 | 3362.1 | -1651.7 | 1710.3 |
| HE | 1977.4 | -1405.2 | 572.3 | 1783.1 | -1611.2 | 171.8 | 3575.2 | -2171.6 | 1403.5 |
| HG | NA | NA | NA | 2012.1 | -1451.6 | 560.5 | 2670 | -1733.2 | 936.8 |
| IN | NA | NA | NA | -62 | 35.2 | -26.9 | 734.8 | 3.7 | 738.5 |
| IP | 1542.2 | -1349.4 | 192.8 | 1870 | -1360.4 | 509.7 | 2940.1 | -1913.9 | 1026.2 |
| MC | 735.1 | -909.9 | -174.8 | 1014.2 | -1091.2 | -77 | 1710.1 | -1324.8 | 385.3 |
| MG | 643.7 | -515.6 | 128.2 | 666.7 | -758.9 | -92.2 | 1185.9 | -751.3 | 434.6 |
| MI | 2282.1 | -1401.4 | 880.6 | 1903.7 | -1320.4 | 583.3 | 4937.3 | -2114.2 | 2823.1 |
| MP | 1667.1 | -912.4 | 754.7 | 2389.9 | -1222.8 | 1167.1 | 3126 | -1343.6 | 1782.5 |
| MR | 3956.1 | -1647.7 | 2308.3 | 2078.7 | -1469.9 | 608.8 | 3574.8 | -1988.4 | 1586.4 |
| NO | 1215.1 | -780.9 | 434.2 | 1822.6 | -1037.5 | 785.1 | 2333.8 | -1349.1 | 984.6 |

^a,b,c^ Changes relative to annual baseline heat-related and cold-related, and total temperature-related years of life lost from ischemic heart disease is 3 200 years, 5 508 years, and 8 708 years, respectively. The unit of change of years of life lost is year.

**Table S****5.** The changes of annual heat-related, cold-related, and net total temperature-related years of life lost from ischemic heart disease for each global-scale climate model under representative concentration pathways (RCPs) in 2070s.

| **GCMs** | **RCP2.6** | | | **RCP4.5** | | | **RCP8.5** | | |
| --- | --- | --- | --- | --- | --- | --- | --- | --- | --- |
|  | **Heat^a^** | **Cold^b^** | **Total^c^** | **Heat^a^** | **Cold^b^** | **Total^c^** | **Heat^a^** | **Cold^b^** | **Total^c^** |
| AC | NA | NA | NA | 5006.2 | -1798.4 | 3207.8 | 5642.2 | -2344.6 | 3297.6 |
| BC | 2317.5 | -837.1 | 1480.4 | 2665.5 | -1185.4 | 1480.1 | 5555 | -1854.3 | 3700.7 |
| CC | 961.1 | -720 | 241.1 | 1934.1 | -1140.9 | 793.2 | 4713.8 | -1896.2 | 2817.5 |
| CE | NA | NA | NA | 8631.9 | -2116.2 | 6515.7 | NA | NA | NA |
| CN | 1166.9 | -593.6 | 573.3 | 1203.2 | -1025.9 | 177.3 | 2599.4 | -1619.5 | 979.9 |
| GD | 97.3 | 43.8 | 141.1 | 760 | -811.2 | -51.2 | NA | NA | NA |
| GF | 1894.3 | -1336 | 558.3 | 3290.3 | -1506.9 | 1783.5 | 9181.7 | -2827.9 | 6353.8 |
| GS | 537.6 | -687.8 | -150.2 | 1326.7 | -1266.9 | 59.8 | 2410.2 | -1880.6 | 529.6 |
| HD | 1401.8 | -1392 | 9.9 | 2429.4 | -2020.2 | 409.2 | 5701.7 | -2745.4 | 2956.2 |
| HE | 1508 | -1382.9 | 125.1 | 3528.2 | -1868.3 | 1659.9 | 7364 | -2899.6 | 4464.5 |
| HG | NA | NA | NA | 1881.3 | -1748 | 133.3 | 7750.9 | -2697.7 | 5053.1 |
| IN | NA | NA | NA | -194.7 | 90.3 | -104.3 | 874 | -921.8 | -47.9 |
| IP | 1014.4 | -1266.9 | -252.5 | 2421.4 | -1750.9 | 670.5 | 6169.9 | -2505.1 | 3664.8 |
| MC | 1612.9 | -1000.6 | 612.3 | 1451.4 | -1328.6 | 122.8 | 3796.6 | -1961.3 | 1835.2 |
| MG | 426.7 | -601.4 | -174.7 | 1300.4 | -967.4 | 333 | 3326.9 | -1909.8 | 1417.1 |
| MI | 3019.8 | -1345.2 | 1674.6 | 4258 | -1790.6 | 2467.4 | 7864.9 | -2688.3 | 5176.7 |
| MP | 1029.4 | -662.2 | 367.2 | 2530.8 | -1227.9 | 1302.9 | 5659.6 | -2060.3 | 3599.3 |
| MR | 2373.9 | -1555.6 | 818.3 | 5113.7 | -2252.6 | 2861.1 | 10817.7 | -3110.6 | 7707.2 |
| NO | 1279.3 | -1385.7 | -106.4 | 2344.9 | -1566.3 | 778.6 | 5305.4 | -2090.1 | 3215.3 |

^a,b,c^ Changes relative to annual baseline heat-related and cold-related, and total temperature-related years of life lost from ischemic heart disease is 3 200 years, 5 508 years, and 8 708 years, respectively. The unit of change of years of life lost is year.

(a)

(b)

**Figure S1.** Sensitivity analyses of exposure-response curve between the daily maximum temperature and years of life lost from ischemic heart disease in the elderly in Tianjin, China, from 2006-2011.

a. Using 4 degrees of freedom of relative humidity in the model (the optimal temperature is 23.7 °C)

b. A natural cubic B-spline basis with 5 degrees of freedom for temperature and a maximum lag of 15 days between temperature and YLL with 6 degrees of freedom in the model (the optimal temperature is 23.4 °C)

**Figure S2.** Projected attributable proportions of heat-related, cold-related and total temperature-related years of life lost from ischaemic heart disease in the elderly for 19 global-scale climate models under 3 representative concentration pathways (RCPs).
